# Recruitment and Retention in Remote Research: Learnings From a Large, Decentralized Real-world Study

**DOI:** 10.2196/40765

**Published:** 2022-11-14

**Authors:** Sophia Xueying Li, Ramzi Halabi, Rahavi Selvarajan, Molly Woerner, Isabell Griffith Fillipo, Sreya Banerjee, Brittany Mosser, Felipe Jain, Patricia Areán, Abhishek Pratap

**Affiliations:** 1 Krembil Centre for Neuroinformatics, Centre for Addiction and Mental Health Toronto, ON Canada; 2 Department of Psychiatry University of Washington Seattle, WA United States; 3 Depression Clinical and Research Program, Department of Psychiatry, Massachusetts General Hospital, Harvard Medical School Boston, MA United States; 4 Department of Psychiatry, University of Toronto Toronto, ON Canada; 5 Vector Institute for Artificial Intelligence Toronto, ON Canada; 6 Kings College London London United Kingdom; 7 Department of Biomedical Informatics and Medical Education, University of Washington Seattle, WA United States

**Keywords:** participant recruitment, participant retention, decentralized studies, active and passive data collection, retention, adherence, compliance, engagement, smartphone, mobile health, mHealth, sensor data, clinical research, data sharing, recruitment, mobile phone

## Abstract

**Background:**

Smartphones are increasingly used in health research. They provide a continuous connection between participants and researchers to monitor long-term health trajectories of large populations at a fraction of the cost of traditional research studies. However, despite the potential of using smartphones in remote research, there is an urgent need to develop effective strategies to reach, recruit, and retain the target populations in a representative and equitable manner.

**Objective:**

We aimed to investigate the impact of combining different recruitment and incentive distribution approaches used in remote research on cohort characteristics and long-term retention. The real-world factors significantly impacting active and passive data collection were also evaluated.

**Methods:**

We conducted a secondary data analysis of participant recruitment and retention using data from a large remote observation study aimed at understanding real-world factors linked to cold, influenza, and the impact of traumatic brain injury on daily functioning. We conducted recruitment in 2 phases between March 15, 2020, and January 4, 2022. Over 10,000 smartphone owners in the United States were recruited to provide 12 weeks of daily surveys and smartphone-based passive-sensing data. Using multivariate statistics, we investigated the potential impact of different recruitment and incentive distribution approaches on cohort characteristics. Survival analysis was used to assess the effects of sociodemographic characteristics on participant retention across the 2 recruitment phases. Associations between passive data-sharing patterns and demographic characteristics of the cohort were evaluated using logistic regression.

**Results:**

We analyzed over 330,000 days of engagement data collected from 10,000 participants. Our key findings are as follows: first, the overall characteristics of participants recruited using digital advertisements on social media and news media differed significantly from those of participants recruited using crowdsourcing platforms (Prolific and Amazon Mechanical Turk; *P*<.001). Second, participant retention in the study varied significantly across study phases, recruitment sources, and socioeconomic and demographic factors (*P*<.001). Third, notable differences in passive data collection were associated with device type (Android vs iOS) and participants’ sociodemographic characteristics. Black or African American participants were significantly less likely to share passive sensor data streams than non-Hispanic White participants (odds ratio 0.44-0.49, 95% CI 0.35-0.61; *P*<.001). Fourth, participants were more likely to adhere to baseline surveys if the surveys were administered immediately after enrollment. Fifth, technical glitches could significantly impact real-world data collection in remote settings, which can severely impact generation of reliable evidence.

**Conclusions:**

Our findings highlight several factors, such as recruitment platforms, incentive distribution frequency, the timing of baseline surveys, device heterogeneity, and technical glitches in data collection infrastructure, that could impact remote long-term data collection. Combined together, these empirical findings could help inform best practices for monitoring anomalies during real-world data collection and for recruiting and retaining target populations in a representative and equitable manner.

## Introduction

### Background

Smartphones offer an unprecedented anytime-anywhere medium for researchers to engage with and assess health-related behaviors in large populations in real-world settings [1,2]. As of 2020, the rate of smartphone ownership in the United States has reached over 80% [3]. The large-scale, high-frequency daily use of such devices coupled with increasingly multimodal onboard sensing capabilities offers an effective approach for conducting large-scale health research [4,5]. The adoption of digital health tools to develop and deploy digitally augmented trials has been rising steadily since the first fully remote decentralized trial in 2011 [6-8]. Recent studies have shown the benefits of remote monitoring using smartphones for assessing real-world behavior [9,10], for managing chronic pain [11], cancer care [12], diabetes [13], Parkinson symptom severity [14], and cardiovascular health [15] and for the delivery of remote interventions [16]. The COVID-19 pandemic has further accelerated this growth, enabling over 220 digitally augmented trials in 2021 alone [17,18].

Using smartphones for health research can also help achieve operational efficiency by relying less on traditional research facilities or intermediaries for data collection, which require in-person contact between the study participants and the research team [6,19,20]. Researchers can communicate asynchronously and synchronously with participants and assess their health by actively and passively collecting individualized real-world data [4,21,22]. Active data are defined as data generated through effortful participation (eg, completing a survey). In contrast, passive data are collected without direct input from participants (eg, the number of daily steps estimated through onboard sensors) [23]. Such scalable remote observational models [6,20] could help investigators to understand people’s day-to-day experiences of living with a health condition [4] and the relationship between individualized real-world behavior and health outcomes [22].

### Challenges in Remote Participant Recruitment and Retention

However, despite the promise of decentralized health research, several challenges related to the representation and inclusiveness of recruitment and the retention of target populations have surfaced [21,24,25], resulting in sparse, unbalanced, and nonrepresentative real-world data collection [21]. Typically, decentralized studies recruit from various web-based sources such as social media (Facebook [26] and Reddit [27]), crowdsourced platforms (Prolific [28]; Amazon Mechanical Turk, MTurk [29]; Centiment [30]; and CloudResearch [31]), and partnerships with patient registries or advocacy groups [32,33]. Although these recruitment channels have shown the potential to reach and recruit large populations remotely [34-36], the long-term and uniform retention of remote participants has been challenging. Recent findings show that retention rates vary from 1% to 50% [24], with monetary incentives being able to significantly improve long-term retention [10]. With large-scale open recruitment approaches, including the use of financial incentives, the risk of enrolling gamers or malicious actors increases [37].

With large studies using multiple web-based sources to reach and recruit participants remotely, there is a need to assess the impact that such strategies have on the characteristics of the enrolled cohorts and their retention in the studies. In addition, further research is needed to understand how variations in study participation incentives (eg, time and frequency of payments) and differences between Android and iOS operating systems [38] affect long-term data collection in decentralized studies.

### Objectives

To investigate some of these challenges in collecting health data through smartphones in real-world settings, we examined the recruitment, retention, and passive data-sharing patterns of more than 10,000 participants in a large, decentralized research study. Specifically, we evaluated the following three key questions: (1) Does combining different recruitment and incentive distribution approaches lead to a heterogeneous cohort with varying characteristics? (2) Can the participant retention and uniformity of data collection in remote studies be affected by cohort heterogeneity? (3) What are the factors that can affect passive data collection in real-world settings?

## Methods

### Ethics Approval

This study was approved and monitored by the Institutional Review Board of the University of Washington (STUDY00004997) and the Department of Defense Human Research Protection Office; the approval for the study was granted on February 11, 2020.

### Study Overview

The participants in the Warfighter Analytics Using Smartphones for Health (WASH) study were volunteers who lived in the United States and agreed to engage in a 12-week smartphone-based study. The primary goal of the study was to understand the real-world factors that could help with the early prediction of cold, influenza, and the impact of traumatic brain injury on daily functioning. The eligible participants were individuals aged ≥19 years, English speakers, residents of the United States, owners, and primary users of iPhone or Android smartphones with internet access. The potential participants were required to complete an eligibility screener before consenting, and those who did not meet the inclusion criteria were not permitted to complete subsequent procedures.

### Recruitment

**Figure 1:**
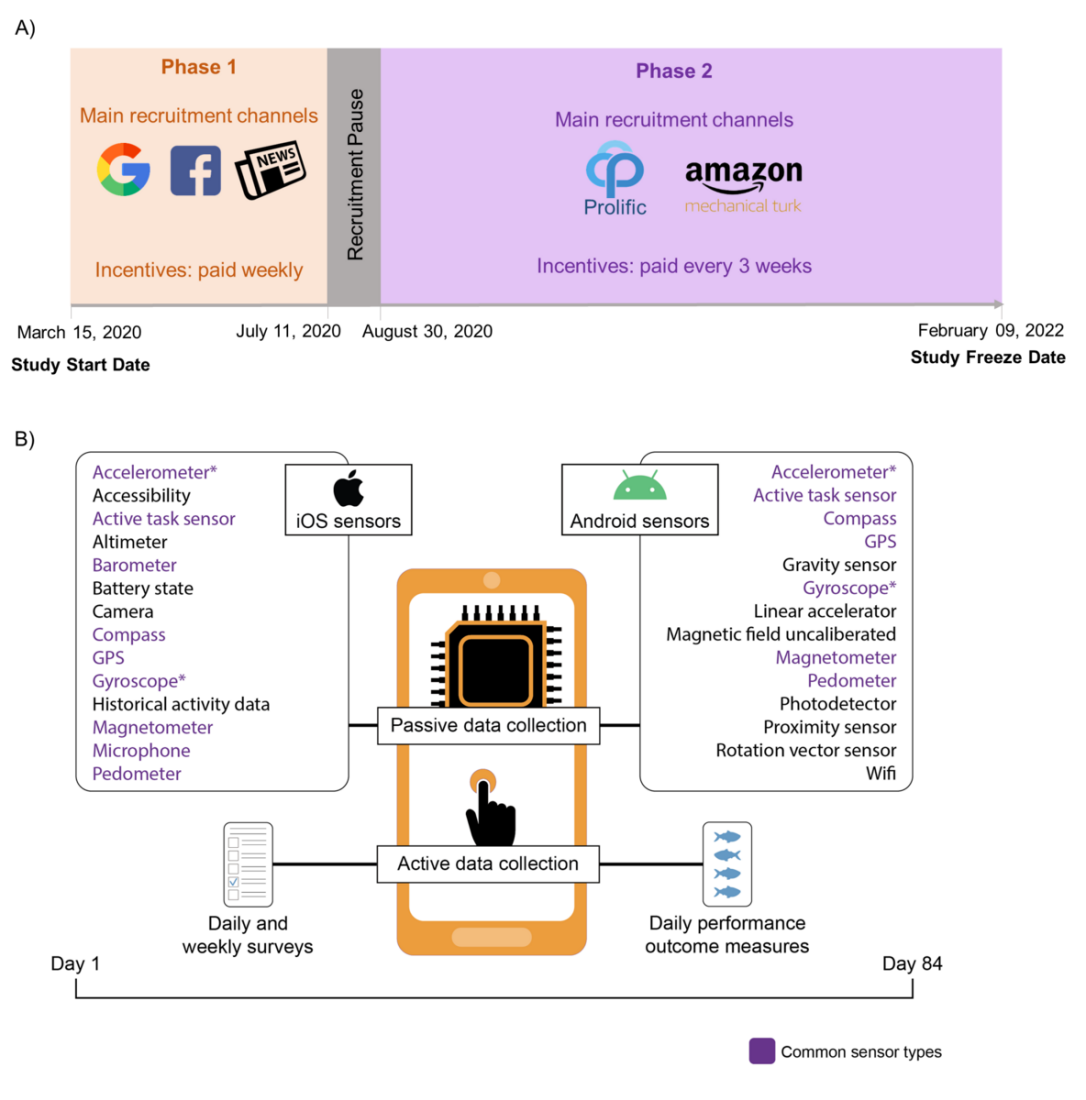
(A) Schematic representation of different study recruitment and participation incentive distribution approaches during phases 1 and 2. Participants recruited during phase 1 were paid weekly (12 times) starting their first day in the study. Anyone who had participated up to October 3, 2020 (who was recruited close to the recruitment pause date) still received weekly payments. Participants recruited during phase 2 were paid every 3 weeks (a total of 4 times) starting their first day in the study. (B) Details of smartphone-based active and passive data collected through the study app during the study observation period (84 days). *Indicates that sharing of accelerometer and gyroscope was made mandatory on August 28, 2020.

Participant recruitment started on March 15, 2020, with rolling enrollment until January 4, 2022. The participants for the study were recruited in 2 phases, using different recruitment and incentive distribution approaches (Figure 1). Participants could receive up to US $90 for completing the baseline survey and 12 weeks of follow-up surveys. The final participation incentive was determined on the basis of the number of complete surveys. Participants were not informed about the financial breakdown during the consent process; however, additional details regarding when they would receive compensation and how much compensation they would receive were provided upon request.

### Participation Incentives

#### Phase 1 (March 15, 2020, to July 11, 2020)

Participants were primarily recruited by placing advertisements on social media platforms that directed potential participants to a study recruitment website. Press releases in local news outlets also served as a recruitment source [39,40]. Participants recruited during this phase were paid weekly on the basis of the days a participant completed all daily surveys. The amount received per day increased throughout the 12 weeks (eg, approximately US $4 in weeks 2 to 4, approximately US $6 in weeks 5 to 8, and US $7 in weeks 9 to 11, with more significant payments made for weeks 1 and 12 because of higher incentives rewarding those who completed the baseline survey in week 1 and the exit survey in week 12). However, a significant increase in study enrollment in June 2020, which seemed to be inconsistent with planned recruitment, led the study team to pause enrollment on July 11, 2020. The analysis of participant activity during this period indicated that some malicious actors were engaged in the study. Further details on the assessment of malicious actors can be found in the study by Bracken et al [37].

#### Phase 2 (August 30, 2020, to the Data Freeze Date, February 9, 2022)

Recruitment resumed on August 30, 2020, after implementing additional strategies to stop fraudulent attempts to join the study, such as disallowing the autofilling of surveys in the study app, changing recruitment sources, and changing incentive payment frequency [37]. Participants were recruited from 2 web-based recruitment platforms, Prolific and MTurk, from January 4 to December 28, 2021, and from May 15 to December 21, 2021. Prolific is a web-based research platform that includes several safeguards for preserving data quality [41-44]; minimizes gamers or malicious actors; and has been shown to be reliable, efficient, and affordable for remote data collection for behavioral research [45]. Similar to Prolific, MTurk is another web-based crowdsourcing platform regularly used in health research to recruit study participants to complete tasks such as data processing, problem-solving, and surveys [46]. In phase 2, participants were paid every 3 weeks, with the first participation incentive payment taking up to 5 weeks. The change in the payment schedule was implemented for allowing sufficient time to execute procedures intended to identify malicious actors.

### Active Surveys

Assessments were divided into 1 longer baseline survey and brief daily assessments. The baseline survey assessing participants’ health history, mood, physical activity, and phone use was administered 24 hours after consent was obtained in phase 1 of the study. However, in phase 2, the baseline health survey was administered immediately after consent was obtained. In both phases, the participants were administered the same scheduled health-related surveys twice daily for 12 weeks. The survey asked participants about their mood, physical activity, and phone use.

### Sensor-Based Data Collection

Sensor-based data were collected actively and passively from participants through the study app. Participants completed performance outcome measures [47] such as standing and walking tests and sharing voice recordings. The participants were also asked to allow the study app to collect passive data from their smartphones. Passive data included, but were not limited to, device movement and orientation; actual and relative location; the device’s status (eg, active use or connected to a data network); and local environmental information such as ambient light, temperature, and humidity. Participants had the option to not share the passive data and remain in the study. However, all participants enrolled in the study on or after August 28, 2020 (before the start of phase 2), were required to allow the study app to passively collect the accelerometer and gyroscope sensor data from their smartphones.

### Data Access

#### Overview

All the data collected from the participants were deidentified. The data collected through the app were encrypted on the phone and stored on secure servers, separate from any identifiable information. Raw data, such as image, proximity, voice, and actual location data of participants, were stored separately from all other sensor data and were not shared with the research team. For this study, data from the enrolled participants between the study launch date (March 15, 2020) and the data freeze date (February 9, 2022) were used for analysis.

#### Data Cleaning

Before analysis, data from 6788 suspected malicious actors were removed based on the rules for flagging such actors that were defined in the study by Bracken et al [37]. Test data collected before the study launch date on March 15, 2020, were removed. If a survey was submitted more than once, we used the most recent submission to assess the participant’s compliance in the study. If participant responses had values outside the expected range of valid values, they were marked as invalid data.

#### Data Harmonization

To investigate participant retention in the study, we classified the data collected by the study app into two broad categories: (1) survey data, representing any active survey data shared by participants through the study app, and (2) sensor data, representing passive continuous sensor data gathered by the study app without active input from participants as well as active sensor data collected during a performance outcome assessment (eg, walking test data collected from accelerometers and gyroscopes).

### Statistical Analysis

#### Overview

Statistical analyses were performed using data from 10,768 participants after excluding 6788 malicious actors from the data set (6788/17,556, 38.66%). Descriptive analyses of recruitment and cohort characteristics for categorical variables were based on frequencies and percentages. Levels of categorical baseline variables that contained <5% of the cohort were omitted or combined with other levels that contained <5% of the cohort to reduce data sparsity in the analysis. We used median values with the 25th and 75th percentile (IQR) for summarizing continuous variables that were not normally distributed. The differences in cohort characteristics were compared using bivariate analysis methods. The chi-square test was used for testing statistically significant differences between categorical variables; the Fisher exact test was used when table cell counts were <5, and the Mann-Whitney *U* test was used for continuous variables. We used the logistic regression model to assess any statistically significant association between patterns of passive data sharing and participants’ sociodemographic characteristics and technical variables. These included race, ethnicity, age, sex, education level, income level, device type, and recruitment phase. Specifically, we compared 3 data-sharing patterns of participants sharing at least 25% (2/8), 50% (4/8), or 75% (6/8) of the 8 common passive data streams between Android and iOS devices. The 95% CIs and *P* values were computed using a Wald *Z* distribution approximation.

We adjusted *P* values by using false discovery rate correction to correct for multiple comparisons across different sensor types. The analyses were conducted using R (version 4.1.1). Statistical significance was assumed when the false discovery rate–corrected *P* value was <.05.

#### Retention Analysis

To examine overall retention in the study, we used the univariate Kaplan-Meier survival curves [48], which were tested for statistically significant differences using the nonparametric log-rank test [49]. A participant’s last day in the study was determined by the last day of their data sharing. To assess the difference in retention between active and passive data sharing, we also computed study retention for active and passive data streams separately. We used right-censored data for the Kaplan-Meier estimator, given that participants could have continued to use the study app beyond the end of the study period (84 days).

To assess the joint effect of multiple variables of interest, including sociodemographics, on participants’ retention in the study, we initially used a multivariate Cox proportional hazards (CoxPH) model [50]. However, one of the key assumptions for CoxPH models (the effect of covariates should not change over time) tested using the Schoenfeld individual test was not met [51]. Multimedia Appendix 1 presents test statistics showing that the CoxPH model assumption is not being met. With the underlying retention data not supporting the CoxPH model assumption, we used a nonparametric log-rank test [52] to assess the statistically significant impact of individual variables on retention within each phase. We cross-compared the median retention for each level of a variable of interest across the 2 study phases.

## Results

### Recruitment

**Figure 2:**
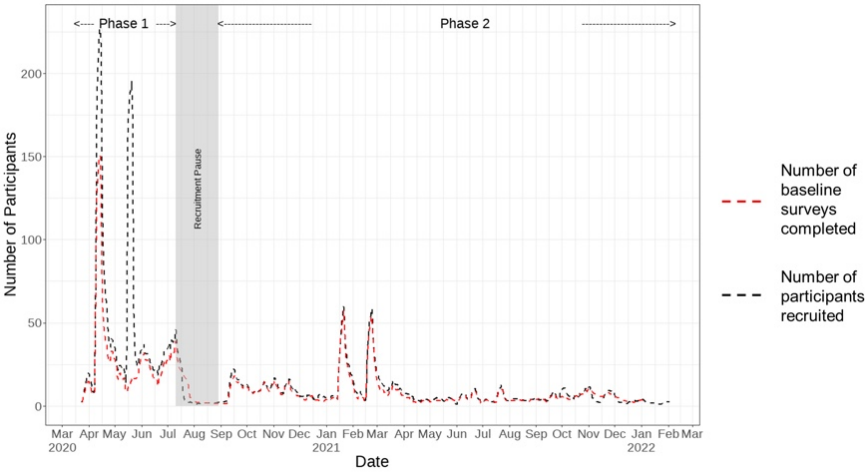
Comparison of the 7-day moving average between the number of participants recruited (black) and the number of baseline surveys completed (red) during the study period. Gray shaded area shows the study recruitment pause phase from July 11, 2020, to August 29, 2020.

As of the data freeze date (February 9, 2022), the study recruited 10,768 participants. Most participants (6494/10,768, 60.3%) were recruited during phase 1, and the remaining (4274/10,768, 39.69%) were recruited during phase 2 (see the Methods section). A significant proportion of participants, most notably in phase 1, did not complete the baseline survey (phase 1: 3135/6494, 48.27%, vs phase 2: 918/4274, 21.47%). Figure 2 compares the recruitment rate of the study with the baseline survey submission rate over time. The number of baseline surveys completed generally was in line with the number of participants recruited during the study period. Recruitment peaked in mid-April and mid-May 2020 for phase 1 and in mid-January and early March 2021 for phase 2. However, during phase 1, between May and July 2020, the number of baseline surveys completed was significantly lower than the number of recruited participants, which explains the large proportion of missing baseline data in phase 1. We further assessed the effect of missing baseline surveys on participant engagement in the study (see the Retention Analysis section). Additional statistics on missingness and invalid data entries in the baseline surveys are summarized in Multimedia Appendix 2.

### Cohort Characteristics

Most of the participants who completed the baseline sociodemographic survey were female (3817/6574, 58.06%). The median age was 30 (IQR 24-40) years, with a larger proportion of participants being aged 19 to 29 years (2949/6267, 47.05%). The non-Hispanic White population was the largest (3938/6677, 58.97%), followed by the Asian (931/6677, 13.94%) and Hispanic or Latino (783/6677, 11.72%) populations. Most participants were iOS users (5883/10,583, 55.58%). Table 1 summarizes the sociodemographic characteristics of the overall cohort.

The population recruited in phase 2 had a higher proportion of younger adults (aged 19 to 29 years; 1685/3194, 52.75%) and a lower proportion of older adults (aged ≥60 years; 94/3194, 2.94%) than that recruited in phase 1 (*P*<.001; Table 1). A higher proportion of Black or African American participants were recruited in phase 2 (phase 1: 267/3342, 7.98%; phase 2: 456/3339, 13.65%; *P*<.001). Notably, a larger proportion of participants (1942/3308, 58.71%) with lower levels of annual income (≤US $49,999) were recruited in phase 2 than in phase 1 (1062/2483, 42.77%; *P*<.001). The proportion of Android versus iOS users also varied across the recruitment phases. A significantly higher proportion of iOS users (*P*<.001) were recruited in phase 1 (3958/5883, 67.27%) than in phase 2 (1925/5883, 32.72%). Multimedia Appendix 3 further compares the sociodemographic characteristics of Android and iOS users across the 2 recruitment phases.

**Table 1:** Characteristics of the overall study cohort (N=10,768) along with comparison of participants recruited between phase 1 (n=6494) and phase 2 (n=4274).

		Overall cohort	Participants recruited during phase 1	Participants recruited during phase 2	Test statistics, chi-square (*df*)	*P* value (phase 1 vs phase 2)
**Age (years), n (%)**		6267 (58.21)	3073 (47.32)	3194 (74.73)	235.29 (4)	<.001
	19-29	2949 (47.05)	1264 (41.14)	1685 (52.8)	—^a^	—
	30-39	1637 (26.12)	739 (24)	898 (28.11)	—	—
	40-49	804 (12.82)	459 (14.9)	345 (10.8)	—	—
	50-59	490 (7.81)	318 (10.3)	172 (5.4)	—	—
	≥60	387 (6.37)	293 (9.51)	94 (2.96)	—	—
	Missing and invalid data^b^	4501	3421	1080	—	—
**Sex, n (%)**		6574 (61.13)	3304 (50.92)	3270 (76.54)	15.25 (1)	<.001
	Female	3817 (58.11)	1997 (60.41)	1820 (55.73)	—	—
	Male	2757 (41.9)	1307 (39.64)	1450 (44.37)	—	—
	Missing and invalid data^b^	4194	3190	1004	—	—
**Race, n (%)**		6681 (62.03)	3342 (51.57)	3339 (78.15)	101.02 (4)	<.001
	Non-Hispanic White	3938 (58.95)	1953 (58.44)	1985 (59.41)	—	—
	Asian	931 (13.93)	487 (14.67)	444 (13.32)	—	—
	Hispanic, Latino, or Spanish	783 (11.72)	424 (12.75)	359 (10.81)	—	—
	Black or African American	723 (10.82)	267 (8.02)	456 (13.77)	—	—
	Other	306 (4.61)	211 (6.32)	95 (2.85)	—	—
	Missing and invalid data^b^	4087	3152	935	—	—
**Marital status, n (%)**		6681 (62.03)	3341 (51.42)	3341 (78.21)	134.02 (3)	<.001
	Single	3312 (49.65)	1439 (43.14)	1873 (56.13)	—	—
	Married or in a domestic partnership	2821 (42.22)	1549 (46.47)	1273 (38.12)	—	—
	Divorced	410 (6.11)	275 (8.28)	135 (4.03)	—	—
	Other	138 (2.16)	78 (2.39)	60 (1.81)	—	—
	Missing and invalid data^b^	4087	3153	933	—	—
**Income level (US $), n (%)**		5793 (53.85)	2483 (38.24)	3310 (77.47)	245.48 (4)	<.001
	<25,000	1736 (30.05)	599 (24.11)	1137 (34.42)	—	—
	25,000 to 49,999	1268 (21.91)	463 (18.64)	805 (24.33)	—	—
	50,000 to 74,999	886 (15.37)	349 (14.15)	537 (16.21)	—	—
	75,000 to 99,999	710 (12.33)	343 (13.85)	367 (11.14)	—	—
	≥100,000	1193 (20.62)	729 (29.41)	464 (14.05)	—	—
	Missing and invalid data^b^	4975	4011	964	—	—
**Level of education, n (%)**		6677 (62.04)	3340 (51.43)	3337 (78.11)	35.34 (2)	<.001
	High school or lower	868 (13.09)	448 (13.41)	420 (12.65)	—	—
	College	3881 (58.16)	1827 (54.71)	2054 (61.62)	—	—
	Graduate school	1928 (28.91)	1065 (31.94)	863 (25.93)	—	—
	Missing and invalid data^b^	4091	3154	937	—	—

^a^Not available.

^b^The proportion is based on the number of participants who completed the baseline survey, and missing and invalid data are presented in Multimedia Appendix 2.

### Passive Data Sharing

The number of data modalities that were passively collected by the study app varied across the Android (31 data modalities) and iOS (14 data modalities) operating systems. The variation in the number of passive data modalities available across Android and iOS devices is because of the available onboard sensors and data collection restrictions across the two operating systems [38]. Of the 31 Android passive data streams, 18 (58%) were shared by at least 50% of the Android users across the 2 study phases (Table 2). In contrast, 86% (12/14) of the distinct passive data streams were shared by at least 50% of the participants using iOS devices. Multimedia Appendix 4 summarizes data-sharing proportions per sensor stratified across Android and iOS devices. None of the participants with iOS devices shared passive data from the camera or barometer. Similarly, participants with Android devices did not share any data from some passive data streams, including temperature, camera, and humidity (Multimedia Appendix 4). This variation in passive data sharing could also be linked to the heterogeneity and nonavailability of specific sensors in some devices. It is worth noting that phase 2 of the study required participants to share accelerometer and gyroscope data passively. However, a small yet notable proportion of the cohort recruited in phase 2 did not share accelerometer (503/4089, 12.31%) and gyroscope (856/4089, 20.89%) data.

In addition, across the 8 passive data streams that were common between Android and iOS devices, the participants’ passive data sharing was linked to sociodemographic characteristics and device type. In total, 3 data-sharing patterns of participants sharing at least 2 (25%), 4 (50%), or 6 (75%) of the total 8 passive data streams were tested. Across all 3 data-sharing patterns, Black or African American participants were found to be statistically significantly less likely to share passive sensor data than non-Hispanic White participants (odds ratio [OR] 0.44-0.49, 95% CI 0.35-0.61; *P*<.001). Furthermore, participants sharing ≥75% (6/8) of the passive data streams were more likely to be iOS device users (OR 1.98, 95% CI 1.71-2.28; *P*<.001) and earning more than US $25,000 per year (OR 1.27-1.55, 95% CI 1.06-1.93; *P*<.001). Multimedia Appendix 5 provides further details on the association between participants’ sociodemographic characteristics and passive data sharing.

**Table 2:** Comparison of the impact of individual sociodemographic variables on the median retention (95% CI) of participants (in days) in the Warfighter Analytics Using Smartphones for Health study across 2 phases.

			Phase 1				Phase 2				
			Retention median (95% CI)		*P* value		Retention median (95% CI)			*P* value	
**Data streams**			37 (37-37)		<.001		51 (49-53)			<.001	
	Passive	37 (37-37)				44 (43-46)					
	Active	36 (36-36)				47 (44-49)					
**Baseline data missingness**					<.001						<.001
	Yes	36 (36-37)				59 (57-62)					
	No	37 (37-38)				19 (16-24)					
**Age (years)**					.01						<.001
	19-29	36 (34-37)				59 (55-63)					
	30-39	36 (34-37)				59 (53-64)					
	40-49	37 (36-38)				60 (50-66)					
	50-59	37 (37-38)				69 (61-79)					
	≥60	38 (37-38)				83 (78-N/A^a^)					
**Race or ethnicity**					<.001						.50
	Asian	39 (38-49)				60 (54-69)					
	Black or African American	6 (4-10)				68 (63-72)					
	Hispanic, Latino, or Spanish	20 (14-25)				58 (50-65)					
	Non-Hispanic White	37 (37-38)				57 (54-61)					
	Other	5 (3-17)				55 (35-67)					
**Income level (US $)**					<.001						.56
	<25,000	37 (36-38)				61 (56-65)					
	25,000 to 49,999	34 (31-36)				60 (55-66)					
	50,000 to 74,999	36 (33-37)				62 (56-68)					
	75,000 to 99,999	24 (19-30)				55 (49-64)					
	>100,000	24 (19-30)				55 (50-61)					
**Level of education**					<.001						.001
	High school or lower	5 (4-10)				50 (46-56)					
	College	38 (37-38)				60 (57-64)					
	Graduate school	36 (35-37)				62 (57-67)					
**Device type**					<.001						<.001
	Android	22 (17- 27)				59.5 (56-63)					
	iOS	37 (37-37)				49 (46-52)					

^a^N/A: not available.

### Participant Retention

The median retention time of the overall cohort was 38 days, within the 84-day study observation period. No meaningful difference was observed in cohort retention across the active (median 37 days) and passive (median 38 days) data streams (Multimedia Appendix 6). The sensitivity analysis of participant retention also showed no significant difference in median survival across the active and passive data streams (Multimedia Appendix 7). Consequently, all subsequent retention analyses were conducted by combining the active and passive data streams.

**Figure 3:**
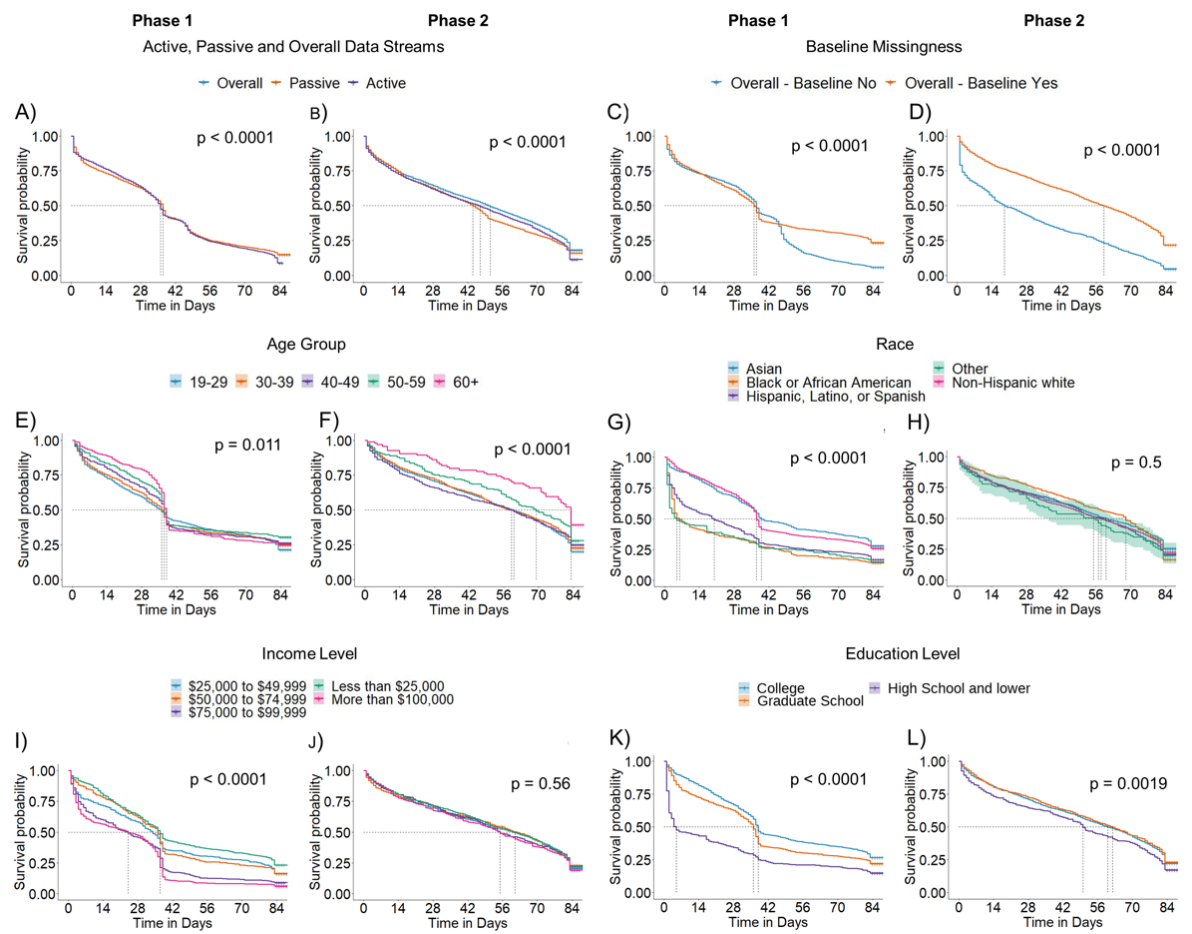
Study retention patterns across the 2 recruitment phases using Kaplan-Meier survival curves. (A)-(B) Cohort retention stratified by active (purple), passive (orange), and overall (ie, active or passive; blue) data streams. (C)-(D) Difference in retention based on completion of the baseline survey; cohort retention by (E)-(F) age group, (G)-(H) race or ethnicity, (I)-(J) income level, and (K)-(L) education level. The shaded region shows the 95% CIs based on the survival model fit.

Notable differences in retention were observed across the population recruited between phases 1 and 2. Participants recruited in phase 2 had a significantly higher median retention (+14 days) than those recruited in phase 1 (phase 1: median 37 days; phase 2: median 51 days; *P*<.001; Figures 3A and 3B). Older participants (≥60 years), recruited in both phases, remained engaged in the study for the longest duration (phase 1 and phase 2 median retention 38 days and 83 days, respectively) relative to the younger cohort (Figures 3E and 3F).

It is worth noting that certain characteristics, including socioeconomic factors, distinctly impacted participant retention across the cohorts recruited in phases 1 and 2 (Table 2). Participants who completed the baseline survey administered immediately after enrollment in phase 2 were retained for a significantly longer period (with median values of baseline survey: yes 59 days vs no 19 days in phase 2; Figure 3D). However, the same trend was not observed for participants recruited from social media platforms in phase 1. Similarly, in phase 1, the non-Hispanic White population was retained in the study for a significantly longer time (median 37 days) than the Hispanic or Latino population (median 20 days; Figure 3G). No meaningful differences were observed among non-Hispanic White and Hispanic, Latino, or Spanish populations in phase 2 (Figure 3H). Education level mainly impacted retention in phase 1. Participants reporting high school or lower education levels had the shortest retention (median 5 days) than other participants (median ≥36 days) in phase 1. Such a large difference in retention because of educational level was not seen in the population recruited in phase 2 from crowdsourcing platforms (Figures 3I-3K). Participants’ self-reported income was also found to be significantly associated with retention in phase 1 only. Participants with incomes of <US $49,999 were retained longer than participants earning >US $100,000 (phase 1: US $49,999 vs US $100,000 median retention 34 days vs 24 days, respectively; *P*<.001; Figures 3I and 3J). We also noticed a dramatic difference in median participant retention between Android and iOS users enrolled in phase 1 (iOS 37 days and Android 22 days; *P*<.001). Table 2 and Multimedia Appendix 8 provide additional results and details on the survival analysis.

## Discussion

### Principal Findings

Our results from the analysis of over 330,000 days of engagement data collected from over 10,000 participants in real-world settings showed that combining different recruitment and incentive distribution approaches can yield heterogeneous cohorts. To the best of our knowledge, this is one of the first studies to empirically assess real-world differences in participants’ sharing of multimodal passive data collected from iOS and Android devices using a bring your own device (BYOD) approach.

Overall, there were 5 key learnings. First, recruiting participants using different media, for example, digital advertisements on social media and web-based newspapers or crowdsourcing platforms, could result in heterogeneous subcohorts with varying characteristics. Second, participant engagement could vary significantly based on the recruitment source (eg, social media vs crowdsourced platforms) and incentive distribution approaches. Third, passive data collection could be substantially affected by technical variations in Android and iOS devices and the sociodemographic demographics of the cohort. Fourth, there is a greater likelihood of participants completing baseline health surveys if they are administered immediately after consent or enrollment. Fifth, monitoring patterns in real-world data collection at the study level could reveal technical glitches that could help guide contextual data filtering and cohort selection, leading to more reliable evidence generation. We now contextualize our principal findings to inform strategies to recruit, retain, and monitor trends in remote data collection to help collect real-world health data in a representative and equitable manner.

#### Combining Recruitment Platforms Could Yield Heterogeneous Real-world Cohorts

Notable differences were observed between the demographic and socioeconomic characteristics of participants recruited from web-based advertisements in social media and newspapers (phase 1) versus crowdsourcing platforms (phase 2). This indicates that combining multiple web-based recruitment sources could yield heterogeneous cohorts, resulting in nonuniform data collection. Future remote studies should assess the potential impact of combining the real-world data obtained from participants enrolled through different recruitment media. Furthermore, the web-based advertisement–based open enrollment approach in phase 1 while successfully recruiting a large cohort quickly also resulted in a significant proportion of bad actors joining the study to receive monetary incentives [37]. On the other hand, crowdsourcing platforms (MTurk and Prolific) were slower in recruiting participants, but their retention was notably higher than that of participants recruited using social media advertisements in phase 1. Indeed, as we have noted in an earlier paper, news outlets and social media recruitment are more likely to attract malicious actors [37] and, as we demonstrate here, less-committed research participants. However, despite the benefits of paid crowdsourcing platforms in effectively reaching and recruiting participants, researchers should carefully consider other factors that could influence the findings of a study [53-57] when recruiting participants from such platforms. These include (1) the primary motivation to remain engaged in remote studies, which may be tied to monetary incentives linked to task completion, and (2) the recruited population may not be representative of the general population [58] or of target health conditions. The characteristics of recruited participants may also vary substantially across recruitment platforms. (3) Nonnaivety-recruited people could be routine participants in research, which could impact the assessment of the actual underlying effect and (4) assessment of the fitness for the purpose of crowdsourced workers for a particular task or study [59-61].

#### Changes in the Incentive Distribution Can Have an Impact on Recruitment and Retention

By increasing the interval at which participants were paid, a significant reduction was observed in the number of malicious actors joining the study in phase 2. Furthermore, keeping the total incentive paid the same, participants who received less compensation weekly (phase 1) remained engaged in the study for a significantly shorter duration than those receiving a larger sum every 3 weeks (phase 2). Although higher retention in phase 2 cannot solely be attributed to a change in incentive distribution (because of a lack of randomization), it is indicative of a potentially interesting behavioral economics model [62] that addresses the perceived burden of participants with episodic but more significant rewards. The value of incentives relative to the study burden also varied by socioeconomic characteristic. In both phases, participants in lower-income groups engaged for longer, likely driven by the incentives, than those in higher-income groups, a finding evident in other studies [63]. Past research has shown that incentives can be an effective way to retain such participants, as small incentives could constitute a way of dealing with monetary barriers [64]. However, researchers should use such incentive-based engagement strategies in a noncoercive manner [65,66] so that potential study participants are not unduly influenced to join and share their data in a research study.

#### Assessing Patterns in Real-world Data Collection Can Reveal Underlying Technical Issues

The evaluation of day-to-day study-level data revealed several patterns indicating transient technical glitches in data collection that, if unaddressed, could bias downstream evidence generation. First, a significant drop in the relative rate of enrollment for baseline survey completion was observed in phase 1 (Figure 2). This could be indicative of a technical glitch in the data collection system or an attempt by a large number of bad actors to join the study to gain monetary incentives (if applicable). Second, active and passive data collection patterns varied notably across the study recruitment phases. For example, we identified 2 periods during the second phase of the study, when the study app collected no passive data despite the completion of active tasks by participants (Multimedia Appendix 9). This is likely a technical glitch in passive data collection that could severely impact the passive data density for the participants who were active during this period. Understanding the context and period in which the data are missing can guide cohort and data selection for a reliable and unbiased downstream analysis. Third, a small but substantial subset of participants recruited in phase 2 did not share the 2 mandatory passive data streams, accelerometer (503/4089, 12.3%) and gyroscope (856/4089, 20.9%), but continued to remain active in the study (Table 2). Near real-time comparison of data modalities shared by participants can help the study teams triage participants who do not meet the required inclusion criteria per the approved study protocol. Fourth, the retention analysis stratified by technical variables (eg, device type) revealed latent idiosyncratic patterns. We observed a notable trend in retention for the participants recruited in phase 1 (Multimedia Appendix 8; Figure 3G). Participants with iOS devices showed a dramatic drop in retention around day 37 compared with a gradual decline for those with Android devices. There may be several plausible reasons for this significant yet idiosyncratic retention pattern, seen only in phase 1 of the study. The sociodemographic characteristics varied significantly between the iOS and Android cohorts in phase 1 compared with phase 2 (Multimedia Appendix 3). In addition, there could have been a bug in the iOS app around week 6 (days 35-42) that could have impacted participant experience and data sharing in phase 1. Taken together, these findings show an urgent need to prioritize real-time monitoring of data collection in real-world settings while the study is in progress. This also provides a just-in-time intervention opportunity to understand, document, and fix the root cause, preventing lower-quality data collection.

#### Passive Data Collection Can Vary Substantially in BYOD Studies

Passive data collected from the participants’ own devices showed that the onboard sensors available across Android and iOS devices can vary substantially. Even for the common passive data streams available on both Android and iOS devices, there can be substantial differences in the sharing of multiple passive data streams linked to participants’ sociodemographic characteristics and device types. For example, in this study, Black individuals or African Americans were significantly less likely to share multimodal passive sensor data (Multimedia Appendix 5). Researchers should expect a high degree of heterogeneity in passive sensor data streams in large BYOD studies and consider the impact of device heterogeneity on data collection, analysis, and evidence generation [38,67-70].

#### Impact of Participants’ Sociodemographic Characteristics on Retention

Older participants (aged ≥60 years) were retained in the study for the longest duration. This finding is consistent with a previous large cross-study comparison of retention [63]. However, the impact of sociodemographic characteristics on participant retention was considerably different between the cohorts recruited using social media advertisements (phase 1) versus crowdsourcing platforms (phase 2). The relative difference in median retention within individual categories (eg, non-Hispanic White vs Hispanic or Latino) was remarkably higher and aligned with prior research [63] in the phase 1 cohort than the cohort recruited in phase 2 (Table 2). This indicates a significant discrepancy in how sociodemographic characteristics may affect participant retention based on recruitment sources. Our findings offer evidence that the population recruited from web-based crowdsourcing platforms shows more homogeneous engagement in research studies than the general population, a behavior that is likely driven by underlying motivation and monetary incentives.

In addition, the demographic composition of the United States is becoming increasingly multiethnic and pluralistic, and it is projected that there will be no majority racial or ethnic groups by 2060 [71]. The sociodemographic characteristics of the enrolled cohort together with nonuniform participant attrition show that large observational studies may not enroll and collect health outcomes from a diverse and representative population uniformly. Future studies should emphasize enrolling diverse populations, such as an All of Us cohort [72], and retaining a diverse sample throughout the study period to ensure that their learnings apply to diverse populations. In addition, some of the challenges in recruiting a diverse cohort have been identified to be related to participants’ perceptions, trust, and willingness to enroll and share their data with researchers, governments, and academic institutions [46,71].

#### Timing of Administration of Baseline Surveys May Impact Completion Rates

The engagement data showed that the timing of administration of the baseline survey could be linked to survey completion rates. The missingness rates of the baseline survey were notably different between the 2 phases (phase 1: 3135/6494, 48.27%; phase 2: 918/4274, 22.47%; Figures 3C and 3D). This indicates that participants were more likely to complete the baseline assessments if they were administered immediately after consent or enrollment (phase 2). This is likely due to a higher level of engagement when enrolling for the study than at subsequent time points, when attention may be captured by other activities. This finding is aligned with some prior research in which participants were more likely to engage with a mobile health app within 24 hours if prompts were provided when participants are most receptive [73]. Moreover, Bidargaddi et al [73] revealed that the degree of engagement is also influenced by other contexts, such as the time of day and the day of the week. These results could help us understand the importance of time of administering an assessment and its impact on data quality in research studies.

### Limitations and Future Directions

The analysis of participant recruitment and retention data from the WASH study should be interpreted within the context of certain limitations. First, large-scale, fully remote data collection started close to the declaration of the COVID-19 pandemic in the United States, which is known to have changed our behavior and interaction with technology and devices [74]. Indeed, Inverso et al [75] showed higher engagement rates during the COVID-19 pandemic because of an increased reliance on technology during the lockdown. The WASH study began recruitment on March 15, 2020, shortly after the World Health Organization declared COVID-19 a pandemic on March 11, 2020. Therefore, we did not have pre-post pandemic data to account for the potential impact of the pandemic on participant engagement with technology and devices. Second, the original purpose of the WASH study was to use the study app to detect cold and influenza symptoms. Thus, participants were not randomized among different recruitment platforms, incentive distribution frequency, and timing of baseline surveys that varied between phases 1 and 2 of the study. Consequently, our findings are not causal or linked to the impact of 1 factor on participant recruitment and retention between phases. For example, this analysis compares the population characteristics of those recruited from web-based crowdsourcing platforms (phase 2) compared with participants enrolling based on social media and local advertisements (open enrollment phase 1) as a whole. We were not able to explore within-phase recruitment differences; that is, between those recruited from social media versus those recruited from local news media advertisements. This is mainly because of the limited information available in the study data, which does not allow for such differences to be investigated. Further research studies using a randomized design are needed to investigate the impact of individual changes in recruitment and retention strategies and their effectiveness for use in decentralized research. Third, we could not control for the participants’ previous experience in crowdsourcing platforms and research tasks, which can be a confounder [41] depending on the nature of the assessment. Future research studies should assess participants’ prior participation in similar or other research studies to assess any differential impact on primary outcomes. Fourth, in phase 1, participant recruitment via press releases was centered in the Greater Seattle area, which may not be representative of the population of the United States. In addition, because of the high proportion of missingness in the baseline geolocation data, we could not determine the geospatial representativeness of the cohort. Future studies should prioritize collecting high-level geolocation data, such as the state, city, or zip code, to help assess the geospatial representativeness of the study cohort. Fifth, we could not account for all the underlying within-study differences in the outcomes; for instance, the probable technical glitches concerning the steep drop in participant engagement at the participant level on day 36 in phase 1 and fluctuations in sensor data collection or management in phase 2 (Multimedia Appendix 9). These technical issues could have impacted the participants’ willingness to remain engaged and increased the perceived burden of participants who were active in the study at the time of technical glitches. Sixth, despite our filtering out bad actors, some could still have been successfully enrolled by creating multiple accounts or using multiple devices. We suggest that future digital health research studies specifically report and compare the impact of different temporal recruitment and incentive strategies on enrolled cohorts’ characteristics and engagement metrics as well as fraudulent enrollments to allow for future replication and the establishment of a set of guidelines for successful methods of participant recruitment and retention.

## Appendix

Multimedia Appendix 1 Test statistics from the Schoenfeld test used to assess the Cox proportional hazards model assumption.

Multimedia Appendix 2 Missing data analysis.

Multimedia Appendix 3 Sociodemographic characteristics of participants in Warfighter Analytics Using Smartphones for Health (WASH) Study stratified by device ownership (iOS and Android).

Multimedia Appendix 4 The proportion of participants who shared sensor data by device type, Android and iOS, across the 2 study phases. *Sensor types that were mandatory to share. **Permission is required to share the data on Android devices.

Multimedia Appendix 5 Odds ratios and 95% CIs from a logistic regression model showing the association between participants’ sociodemographic characteristics and passive data sharing. The results from 3 separate models comparing passive data-sharing patterns using 2 (25%), 4 (50%), and 6 (75%) of the 8 passive data streams that were common between Android and iOS devices are shown below. **P*<.051. ***P*<.001. ****P*<.0001.

Multimedia Appendix 6 Overall participant retention in the Warfighter Analytics Using Smartphones for Health (WASH) study.

Multimedia Appendix 7 Sensitivity analysis of participant retention by extending the observation window (84 days) by 2 weeks.

Multimedia Appendix 8 Additional survival curves.

Multimedia Appendix 9 Patterns in Study App Data Collection.

## Abbreviations

BYOD: bring your own device
CoxPH: Cox proportional hazards
MTurk: Amazon Mechanical Turk
OR: odds ratio
WASH: Warfighter Analytics Using Smartphones for Health

## Data Availability

The individual-level engagement data used for the study will be available upon reasonable request from the corresponding author. The complete code used for data loading and analysis is available through an open-source GitHub code repository [28].

## References

[1] Cao J, Lim Y, Sengoku S, Guo X, Kodama K (2021). Exploring the shift in international trends in mobile health research from 2000 to 2020: bibliometric analysis. JMIR Mhealth Uhealth.

[2] Lunn MR, Lubensky M, Hunt C, Flentje A, Capriotti MR, Sooksaman C, Harnett T, Currie D, Neal C, Obedin-Maliver J (2019). A digital health research platform for community engagement, recruitment, and retention of sexual and gender minority adults in a national longitudinal cohort study--the PRIDE study. J Am Med Inform Assoc.

[3] (2022). Penetration rate of smartphones in selected countries 2021. Statista.

[4] De Brouwer W, Patel CJ, Manrai AK, Rodriguez-Chavez IR, Shah NR (2021). Empowering clinical research in a decentralized world. NPJ Digit Med.

[5] Badawy R, Hameed F, Bataille L, Little MA, Claes K, Saria S, Cedarbaum JM, Stephenson D, Neville J, Maetzler W, Espay AJ, Bloem BR, Simuni T, Karlin DR (2019). Metadata concepts for advancing the use of digital health technologies in clinical research. Digit Biomark.

[6] Van Norman GA (2021). Decentralized clinical trials: the future of medical product development?∗. JACC Basic Transl Sci.

[7] Roehr B (2011). Pfizer launches virtual clinical trial. BMJ.

[8] (2011). Pfizer Conducts First “Virtual” Clinical Trial Allowing Patients to Participate Regardless Of Geography. Pfizer.

[9] Pratap A, Atkins DC, Renn BN, Tanana MJ, Mooney SD, Anguera JA, Areán PA (2019). The accuracy of passive phone sensors in predicting daily mood. Depress Anxiety.

[10] Nickels S, Edwards MD, Poole SF, Winter D, Gronsbell J, Rozenkrants B, Miller DP, Fleck M, McLean A, Peterson B, Chen Y, Hwang A, Rust-Smith D, Brant A, Campbell A, Chen C, Walter C, Arean PA, Hsin H, Myers LJ, Marks WJ, Mega JL, Schlosser DA, Conrad AJ, Califf RM, Fromer M (2021). Toward a mobile platform for real-world digital measurement of depression: user-centered design, data quality, and behavioral and clinical modeling. JMIR Ment Health.

[11] Lee JL, Cerrada CJ, Vang MK, Scherer K, Tai C, Tran JL, Juusola JL, Sang CN (2021). The DiSCover project: protocol and baseline characteristics of a decentralized digital study assessing chronic pain outcomes and behavioral data. medRxiv.

[12] Sundquist S, Batist G, Brodeur-Robb K, Dyck K, Eigl BJ, Lee DK, Limoges J, Longstaff H, Pankovich J, Sadura A, Sullivan P, Dancey JE (2021). CRAFT-a proposed framework for decentralized clinical trials participation in Canada. Curr Oncol.

[13] Larbi D, Randine P, Årsand E, Antypas K, Bradway M, Gabarron E (2020). Methods and evaluation criteria for apps and digital interventions for diabetes self-management: systematic review. J Med Internet Res.

[14] Omberg L, Chaibub Neto E, Perumal TM, Pratap A, Tediarjo A, Adams J, Bloem BR, Bot BM, Elson M, Goldman SM, Kellen MR, Kieburtz K, Klein A, Little MA, Schneider R, Suver C, Tarolli C, Tanner CM, Trister AD, Wilbanks J, Dorsey ER, Mangravite LM (2022). Remote smartphone monitoring of Parkinson's disease and individual response to therapy. Nat Biotechnol.

[15] Mayfield JJ, Chatterjee NA, Noseworthy PA, Poole JE, Ackerman MJ, Stewart J, Kissinger PJ, Dwyer J, Hosek S, Oyedele T, Paasche-Orlow MK, Paolino K, Friedman PA, Waters C, Moreno J, Leingang H, Heller KB, Morrison SA, Krows ML, Barnabas RV, Baeten J, Johnston C, Sridhar AR, COVID-19 Early Treatment Team (2021). Implementation of a fully remote randomized clinical trial with cardiac monitoring. Commun Med (Lond).

[16] Badawy SM, Radovic A (2020). Digital approaches to remote pediatric health care delivery during the COVID-19 pandemic: existing evidence and a call for further research. JMIR Pediatr Parent.

[17] Farah J, Vasey J, Kallenbach L, Caplea G (2021). Conducting Research at the Point of Care. Veradigm® Allscripts Healthcare.

[18] Hillman A, Castañeda R (2022). Who and what is at the crest of the clinical trial decentralisation wave?. Clinical Trials Arena.

[19] Inan OT, Tenaerts P, Prindiville SA, Reynolds HR, Dizon DS, Cooper-Arnold K, Turakhia M, Pletcher MJ, Preston KL, Krumholz HM, Marlin BM, Mandl KD, Klasnja P, Spring B, Iturriaga E, Campo R, Desvigne-Nickens P, Rosenberg Y, Steinhubl SR, Califf RM (2020). Digitizing clinical trials. NPJ Digit Med.

[20] Khozin S, Coravos A (2019). Decentralized trials in the age of real-world evidence and inclusivity in clinical investigations. Clin Pharmacol Ther.

[21] Ferrar J, Griffith GJ, Skirrow C, Cashdollar N, Taptiklis N, Dobson J, Cree F, Cormack FK, Barnett JH, Munafò MR (2021). Developing digital tools for remote clinical research: how to evaluate the validity and practicality of active assessments in field settings. J Med Internet Res.

[22] Hilderbrand A, Zangrilli M, Stinson M (2021). Decentralized clinical trial case study: five-stage process for recruiting and completing a site-less clinical study in less time and lower cost than traditional methods. Am J Health Res.

[23] Torous J, Kiang MV, Lorme J, Onnela JP (2016). New tools for new research in psychiatry: a scalable and customizable platform to empower data driven smartphone research. JMIR Ment Health.

[24] Goodday SM, Karlin E, Alfarano A, Brooks A, Chapman C, Desille R, Rangwala S, Karlin DR, Emami H, Woods NF, Boch A, Foschini L, Wildman M, Cormack F, Taptiklis N, Pratap A, Ghassemi M, Goldenberg A, Nagaraj S, Walsh E, Friend S, Stress And Recovery Participants (2021). An alternative to the light touch digital health remote study: the stress and recovery in frontline COVID-19 health care workers study. JMIR Form Res.

[25] Meyerowitz-Katz G, Ravi S, Arnolda L, Feng X, Maberly G, Astell-Burt T (2020). Rates of attrition and dropout in app-based interventions for chronic disease: systematic review and meta-analysis. J Med Internet Res.

[26] Kapp JM, Peters C, Oliver DP (2013). Research recruitment using Facebook advertising: big potential, big challenges. J Cancer Educ.

[27] Shatz I (2016). Fast, free, and targeted: Reddit as a source for recruiting participants online. Soc Sci Comput Rev.

[28] Prolific.

[29] Amazon Mechanical Turk.

[30] Centiment.

[31] CloudResearch. Prime Research Solutions.

[32] PatientsLikeMe.

[33] The Michael J. Fox Foundation for Parkinson's Research.

[34] Myers TL, Augustine EF, Baloga E, Daeschler M, Cannon P, Rowbotham H, Chanoff E, Jensen-Roberts S, Soto J, Holloway RG, Marras C, Tanner CM, Dorsey ER, Schneider RB, 23andMe Research Team (2022). Recruitment for remote decentralized studies in Parkinson's disease. J Parkinsons Dis.

[35] Anguera JA, Jordan JT, Castaneda D, Gazzaley A, Areán PA (2016). Conducting a fully mobile and randomised clinical trial for depression: access, engagement and expense. BMJ Innov.

[36] Sun S, Folarin AA, Ranjan Y, Rashid Z, Conde P, Stewart C, Cummins N, Matcham F, Dalla Costa G, Simblett S, Leocani L, Lamers F, Sørensen PS, Buron M, Zabalza A, Guerrero Pérez AI, Penninx BW, Siddi S, Haro JM, Myin-Germeys I, Rintala A, Wykes T, Narayan VA, Comi G, Hotopf M, Dobson RJ, RADAR-CNS Consortium (2020). Using smartphones and wearable devices to monitor behavioral changes during COVID-19. J Med Internet Res.

[37] Bracken BK, Wolcott J, Potoczny-Jones I, Mosser BA, Griffith-Fillipo IR, Arean PA (2022). Detection and remediation of malicious actors for studies involving remote data collection. Proceedings of the 15th International Joint Conference on Biomedical Engineering Systems and Technologies.

[38] Nishiyama Y, Ferreira D, Sasaki W, Okoshi T, Nakazawa J, Dey AK, Sezaki K (2020). Using iOS for inconspicuous data collection: a real-world assessment. Adjunct Proceedings of the 2020 ACM International Joint Conference on Pervasive and Ubiquitous Computing and Proceedings of the 2020 ACM International Symposium on Wearable Computers.

[39] Boyle A (2020). UW Medicine seeks 25,000 volunteers to try outbreak-predicting smartphone app. GeekWire.

[40] (2020). UW Medicine recruiting for app to predict next outbreak. University of Washington Medicine.

[41] Palan S, Schitter C (2018). Prolific.ac—a subject pool for online experiments. J Behav Exp Finance.

[42] Peer E, Brandimarte L, Samat S, Acquisti A (2017). Beyond the Turk: alternative platforms for crowdsourcing behavioral research. J Exp Soc Psychol.

[43] Why participants get banned. Prolific.

[44] Bradley P (2018). Bots and data quality on crowdsourcing platforms. Prolific.

[45] Lettmann H, Lumsden J (2018). Prolific's participant pool – the present and the future. Prolific.

[46] Pratap A, Allred R, Duffy J, Rivera D, Lee HS, Renn BN, Areán PA (2019). Contemporary views of research participant willingness to participate and share digital data in biomedical research. JAMA Netw Open.

[47] Coravos A, Goldsack JC, Karlin DR, Nebeker C, Perakslis E, Zimmerman N, Erb MK (2019). Digital medicine: a primer on measurement. Digit Biomark.

[48] Rich JT, Neely JG, Paniello RC, Voelker CC, Nussenbaum B, Wang EW (2010). A practical guide to understanding Kaplan-Meier curves. Otolaryngol Head Neck Surg.

[49] Hazra A, Gogtay N (2017). Biostatistics series module 9: survival analysis. Indian J Dermatol.

[50] Kumar D, Klefsjö B (1994). Proportional hazards model: a review. Reliab Eng Syst Saf.

[51] Hess KR (1995). Graphical methods for assessing violations of the proportional hazards assumption in Cox regression. Stat Med.

[52] Bland JM, Altman DG (2004). The logrank test. BMJ.

[53] Pennington CR, Jones AJ, Tzavella L, Chambers CD, Button KS (2022). Beyond online participant crowdsourcing: the benefits and opportunities of big team addiction science. Exp Clin Psychopharmacol.

[54] Khare R, Good BM, Leaman R, Su AI, Lu Z (2016). Crowdsourcing in biomedicine: challenges and opportunities. Brief Bioinform.

[55] Gleibs IH (2017). Are all "research fields" equal? Rethinking practice for the use of data from crowdsourcing market places. Behav Res Methods.

[56] Lovett M, Bajaba S, Lovett M, Simmering MJ (2018). Data quality from crowdsourced surveys: a mixed method inquiry into perceptions of Amazon's Mechanical Turk Masters. Appl Psychol.

[57] Berry C, Kees J, Burton S (2022). Drivers of data quality in advertising research: differences across MTurk and professional panel samples. J Advert.

[58] Walters K, Christakis DA, Wright DR (2018). Are Mechanical Turk worker samples representative of health status and health behaviors in the U.S.?. PLoS One.

[59] Anderson CA, Allen JJ, Plante C, Quigley-McBride A, Lovett A, Rokkum JN (2019). The MTurkification of social and personality psychology. Pers Soc Psychol Bull.

[60] Edgar J, Murphy J, Keating M (2016). Comparing traditional and crowdsourcing methods for pretesting survey questions. SAGE Open.

[61] Tahaei M, Vaniea K (2022). Recruiting participants with programming skills: a comparison of four crowdsourcing platforms and a CS student mailing list. Proceedings of the 2022 CHI Conference on Human Factors in Computing Systems.

[62] Kamenica E (2012). Behavioral economics and psychology of incentives. Annu Rev Econ.

[63] Pratap A, Neto EC, Snyder P, Stepnowsky C, Elhadad N, Grant D, Mohebbi MH, Mooney S, Suver C, Wilbanks J, Mangravite L, Heagerty PJ, Areán P, Omberg L (2020). Indicators of retention in remote digital health studies: a cross-study evaluation of 100,000 participants. NPJ Digit Med.

[64] Galárraga O, Sosa-Rubí SG (2019). Conditional economic incentives to improve HIV prevention and treatment in low-income and middle-income countries. Lancet HIV.

[65] Singer E, Bossarte RM (2006). Incentives for survey participation when are they "coercive"?. Am J Prev Med.

[66] Ambuehl S, Ockenfels A (2016). The ethics of incentivizing the uninformed. A vignette study. SSRN J.

[67] Nishiyama Y, Ferreira D, Eigen Y, Sasaki W, Okoshi T, Nakazawa J, Dey AK, Sezaki K (2020). IOS crowd–sensing won’t hurt a bit!: AWARE framework and sustainable study guideline for iOS platform. Proceedings of the 8th International Conference on Distributed, Ambient and Pervasive Interactions.

[68] Russell C, McCarthy M, Cappelleri JC, Wong S (2021). Choosing a mobile sensor technology for a clinical trial: statistical considerations, developments and learnings. Ther Innov Regul Sci.

[69] Demanuele C, Lokker C, Jhaveri K, Georgiev P, Sezgin E, Geoghegan C, Zou KH, Izmailova E, McCarthy M (2022). Considerations for conducting bring your own "device" (BYOD) clinical studies. Digit Biomark.

[70] Cho PJ, Yi J, Ho E, Shandhi MM, Dinh Y, Patil A, Martin L, Singh G, Bent B, Ginsburg G, Smuck M, Woods C, Shaw R, Dunn J (2022). Demographic imbalances resulting from the bring-your-own-device study design. JMIR Mhealth Uhealth.

[71] Yancey AK, Ortega AN, Kumanyika SK (2006). Effective recruitment and retention of minority research participants. Annu Rev Public Health.

[72] Denny JC, Rutter JL, Goldstein DB, Philippakis A, Smoller JW, Jenkins G, Dishman E, All of Us Research Program Investigators (2019). The "All of Us" research program. N Engl J Med.

[73] Bidargaddi N, Almirall D, Murphy S, Nahum-Shani I, Kovalcik M, Pituch T, Maaieh H, Strecher V (2018). To prompt or not to prompt? A microrandomized trial of time-varying push notifications to increase proximal engagement with a mobile health app. JMIR Mhealth Uhealth.

[74] Vargo D, Zhu L, Benwell B, Yan Z (2021). Digital technology use during COVID-19 pandemic: a rapid review. Human Behav and Emerg Tech.

[75] Inverso H, Abadula F, Morrow T, LeStourgeon L, Parmar A, Streisand R, Jaser SS (2021). Pivoting during a pandemic: lessons learned from transitioning a multisite randomized controlled trial to a remote protocol in response to COVID-19. Transl Behav Med.

