# Supplementary material for: Recruitment and Retention in Remote Research: Learnings From a Large, Decentralized Real-world Study
Source: JMIR Form Res. 2022 Nov 14;6(11):e40765. doi: 10.2196/40765 (PMC9706389; doi:10.2196/40765)
Supplement: Multimedia Appendix 1 [file formative_v6i11e40765_app1.pdf]

Multimedia Appendix 1 - Test statistics from Schoenfeld test to assess the CoxPH model assumption

	Chi-square statistic		p-value	
	**Phase 1**	**Phase2**	**Phase 1**	**Phase2**
**Age**	67.2	2.34	8.8e-14	0.6530
**Gender**	47.0	2.51	7.2e-12	0.1134
**Race**	96.5	26.04	< 2e-16	3.1e-05
**Marital Status**	32.5	2.38	4.2e-07	0.4966
**Income Level**	22.9	3.05	0.00013	0.5502
**Education Level**	52.0	2.35	5.0e-12	0.3082
**Global**	322.6	36.31	< 2e-16	0.0064
