# Supplementary material for: Recruitment and Retention in Remote Research: Learnings From a Large, Decentralized Real-world Study
Source: JMIR Form Res. 2022 Nov 14;6(11):e40765. doi: 10.2196/40765 (PMC9706389; doi:10.2196/40765)
Supplement: Multimedia Appendix 2 [file formative_v6i11e40765_app2.pdf]

Multimedia Appendix 2. Missing data analysis

	WASH Study Full Cohort<br>N = 10768<br>n (%)	WASH Study Cohort<br>Phase 1<br>N = 6494<br>n (%)	WASH Study Cohort<br>Phase 2<br>N = 4274<br>n (%)
**Age (years)**			
Valid and Not missing	6267 (58.2)	3073 (47.3)	3194 (74.7)
Invalid entries	208 (1.9)	100 (1.5)	108 (2.5)
Missing	4293 (39.9)	3321 (51.1)	972 (22.7)
**Gender**			
Valid and Not missing	6697 (62.2)	3351 (51.6)	3346 (78.3)
Invalid entries	1 (0.0)	0	1 (0.0)
Missing	4070 (37.8)	3143 (48.4)	927 (21.7)
**Race**			
Valid and Not missing	6677 (62.0)	3340 (51.4)	3337 (78.1)
Invalid entries	3 (0.0)	0	3 (0.1)
Missing	4088 (38.0)	3154 (48.6)	934 (21.9)
**Marital Status**			
Valid and Not missing	6682 (62.1)	3341 (51.4)	3341 (78.2)
Invalid entries	1 (0.0)	0	1 (0.0)
Missing	4085 (37.9)	3153 (48.6)	932 (21.8)
**Income Level**			
Valid and Not missing	5793 (53.8)	2483 (38.2)	3310 (77.4)
Invalid entries	4 (0.0)	0	4 (0.1)
Missing	4971 (46.2)	4011 (61.8)	960 (22.5)
**Education**			
Valid and Not missing	6677 (62.0)	3340 (51.4)	3337 (78.1)
Invalid entries	4 (0.0)	0	4 (0.1)
Missing	4087 (38.0)	3154 (48.6)	933 (21.8)
