# Supplementary material for: Recruitment and Retention in Remote Research: Learnings From a Large, Decentralized Real-world Study
Source: JMIR Form Res. 2022 Nov 14;6(11):e40765. doi: 10.2196/40765 (PMC9706389; doi:10.2196/40765)
Supplement: Multimedia Appendix 3 [file formative_v6i11e40765_app3.pdf]

Multimedia Appendix 3. Sociodemographic characteristics of WASH iOS and Android Users

	iOS users N = 5883		Android users N = 4700	
	Phase 1 n = 3958 (67.3%)	Phase 2 n = 1925 (32.7%)	Phase 1 n = 2536 (54.0%)	Phase 2 n = 2164 (46.0%)
**Age (years)**				
19-29	786 (45.1)	869 (59.2)	478 (35.9)	760 (47.0)
30-39	358 (20.6)	340 (23.2)	381 (28.6)	530 (32.8)
40-49	236 (13.6)	130 (8.9)	223 (16.7)	200 (12.4)
50-59	174 (10.0)	84 (5.7)	144 (10.8)	80 (5.0)
60+	187 (10.7)	44 (3.0)	106 (8.0)	46 (2.8)
**Gender**				
Female	1260 (71.6)	981 (66.0)	737 (47.7)	783 (47.0)
Male	500 (28.4)	505 (34.0)	807 (52.3)	884 (53.0)
**Race**				
Asian	323 (18.2)	266 (17.6)	164 (10.5)	166 (9.7)
Black or African American	88 (5.0)	95 (6.3)	179 (11.4)	353 (20.7)
Hispanic, Latino, or Spanish	189 (10.6)	151 (10.0)	235 (15.0)	188 (11.0)
Non-Hispanic white	1127 (63.4)	966 (63.8)	826 (52.8)	940 (55.2)
Other	50 (2.8)	37 (2.4)	161 (10.3)	56 (3.3)
**Marital Status**				
Divorced	93 (5.2)	42 (2.8)	182 (11.6)	84 (4.9)
Married/Domestic Partner	790 (44.5)	515 (34.0)	759 (48.5)	702 (41.2)
Single	867 (48.8)	939 (62.0)	572 (36.6)	880 (51.6)
Other	27 (1.5)	19 (1.3)	51 (3.3)	39 (2.3)
**Income Level**				
Less than $25,000	480 (27.6)	544 (36.2)	119 (16.0)	559 (33.1)
$25,000 to $49,999	316 (18.2)	350 (23.3)	147 (19.8)	425 (25.2)
$50,000 to $74,999	275 (15.8)	246 (16.4)	74 (10.0)	264 (15.6)

$75,000 to $99,999	222 (12.8)	156 (10.4)	121 (16.3)	201 (11.9)
More than $100,000	447 (25.7)	206 (13.7)	282 (38.0)	238 (14.1)
**Education**				
High School and lower	121 (6.8)	138 (9.1)	327 (20.9)	271 (15.9)
College	1087 (61.1)	965 (63.7)	740 (47.4)	1007 (59.2)
Graduate School	570 (32.1)	413 (27.2)	495 (31.7)	423 (24.9)
