# Supplementary material for: Recruitment and Retention in Remote Research: Learnings From a Large, Decentralized Real-world Study
Source: JMIR Form Res. 2022 Nov 14;6(11):e40765. doi: 10.2196/40765 (PMC9706389; doi:10.2196/40765)
Supplement: Multimedia Appendix 5 [file formative_v6i11e40765_app5.pdf]

Multimedia Appendix 5 - Odds ratio and 95% confidence intervals (CI) from a logistic regression model showing the association between participants sociodemographics and passive data sharing. Results from three separate models comparing passive data sharing patterns using 2 (25%), 4(50%), and 6(75%) of the eight common passive data streams between Android and iOS devices are shown below.

** p < 0.051 ** p < 0.001 *** p < 0.0001*

Percent of Passive Data Streams Shared (N=8)	25% (N=2)		50% (N=4)		75% (N=6)	
	**Odds Ratio**	**95% CI**	**Odds Ratio**	**95% CI**	**Odds Ratio**	**95% CI**
Intercept	**18.24*****	11.92 - 28.37	**6.90*****	4.95 - 9.69	**1.68*****	1.3 - 2.2
**Study Phase (ref = Phase 1)**						
Phase 2	**0.60*****	0.48 - 0.76	**0.65****	0.53 - 0.77	0.96	0.83 - 1.11
**Device Type (ref = Android)**						
iOS	**0.59*****	0.47 - 0.75	1.01	0.84 - 1.21	**1.98*****	1.71 - 2.28
**Race/Ethnicity (ref = Non Hispanic White)**						
Asian	1.03	0.76 - 1.4	1.08	0.84 - 1.4	0.91	0.75 - 1.12
Black or African American	**0.46*****	0.35 - 0.61	**0.49*****	0.39 - 0.62	**0.44*****	0.36 - 0.53
Hispanic	1.14	0.81 - 1.63	1.04	0.81 - 1.38	**0.68*****	0.56 - 0.83
Other	1.08	0.6 - 2.16	1.57	0.93 - 2.88	0.69	0.49 - 0.97
**Age (ref =**						
30-39	1.28	0.99 - 1.68	1.23	1 - 1.52	1.09	0.92 - 1.29
40-49	1.00	0.72 - 1.41	0.97	0.74 - 1.28	0.98	0.79 - 1.22
50-59	1.55	0.96 - 2.62	1.38	0.95 - 2.06	1.33	0.99 - 1.8

60+	1.49	0.87 - 2.77	1.71	1.07 - 2.9	1.52	1.07 - 2.23
**Gender** (ref = Female)						
Male	0.94	0.75 - 1.16	0.8	0.67 - 0.95	0.85	0.74 - 0.98
Non-binary	0.84	0.45 - 1.76	0.82	0.49 - 1.49	0.98	0.62 - 1.59
**Education** (ref = High School and lower)						
College	1.07	0.76 - 1.47	1.17	0.9 - 1.5	1.37*	1.12 - 1.68
Graduate School	1.10	0.74 - 1.59	1.18	0.88 - 1.59	1.25	0.99 - 1.57
**Income Level** (ref = Less than $25,000)						
$25,000 to $49,999	1.14	0.87 - 1.5	1.14	0.92 - 1.43	**1.27****	1.06 - 1.52
$50,000 to $74,999	1.23	0.89 - 1.72	1.33	1.02 - 1.74	**1.55*****	1.25 - 1.93
$75,000 to $99,999	0.93	0.66 - 1.32	1.18	0.88 - 1.58	**1.44****	1.13 - 1.83
More than $100,000	1.49	1.05 - 2.15	**1.537****	1.16 - 2.05	1.30	1.05 - 1.62
