# Supplementary figures and images for: Recruitment and Retention in Remote Research: Learnings From a Large, Decentralized Real-world Study
Source: JMIR Form Res. 2022 Nov 14;6(11):e40765. doi: 10.2196/40765 (PMC9706389; doi:10.2196/40765)

## Multimedia Appendix 6 - Overall retention

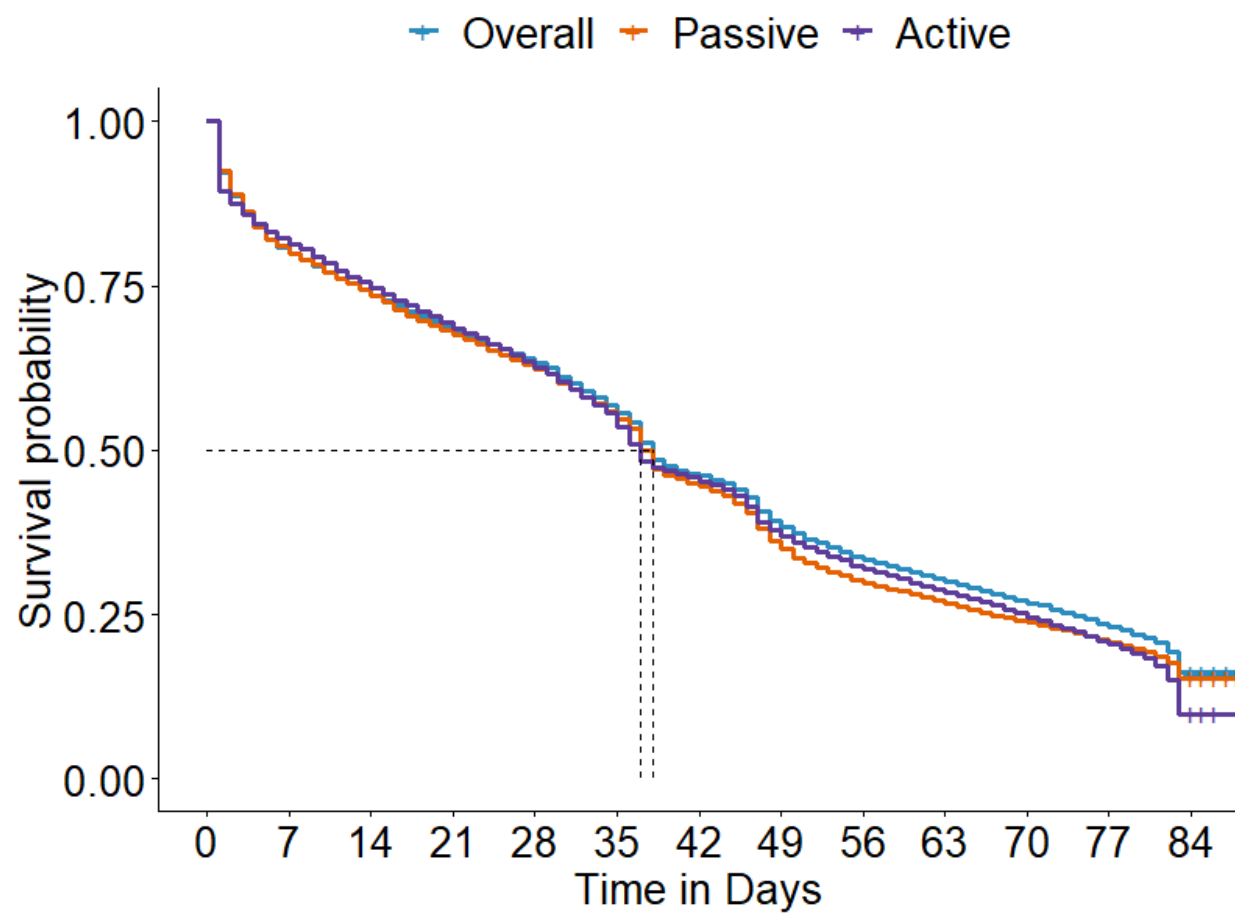

Supplement: Multimedia Appendix 6 [file formative_v6i11e40765_app6.pdf]

## Multimedia Appendix 8 - Additional survival curves

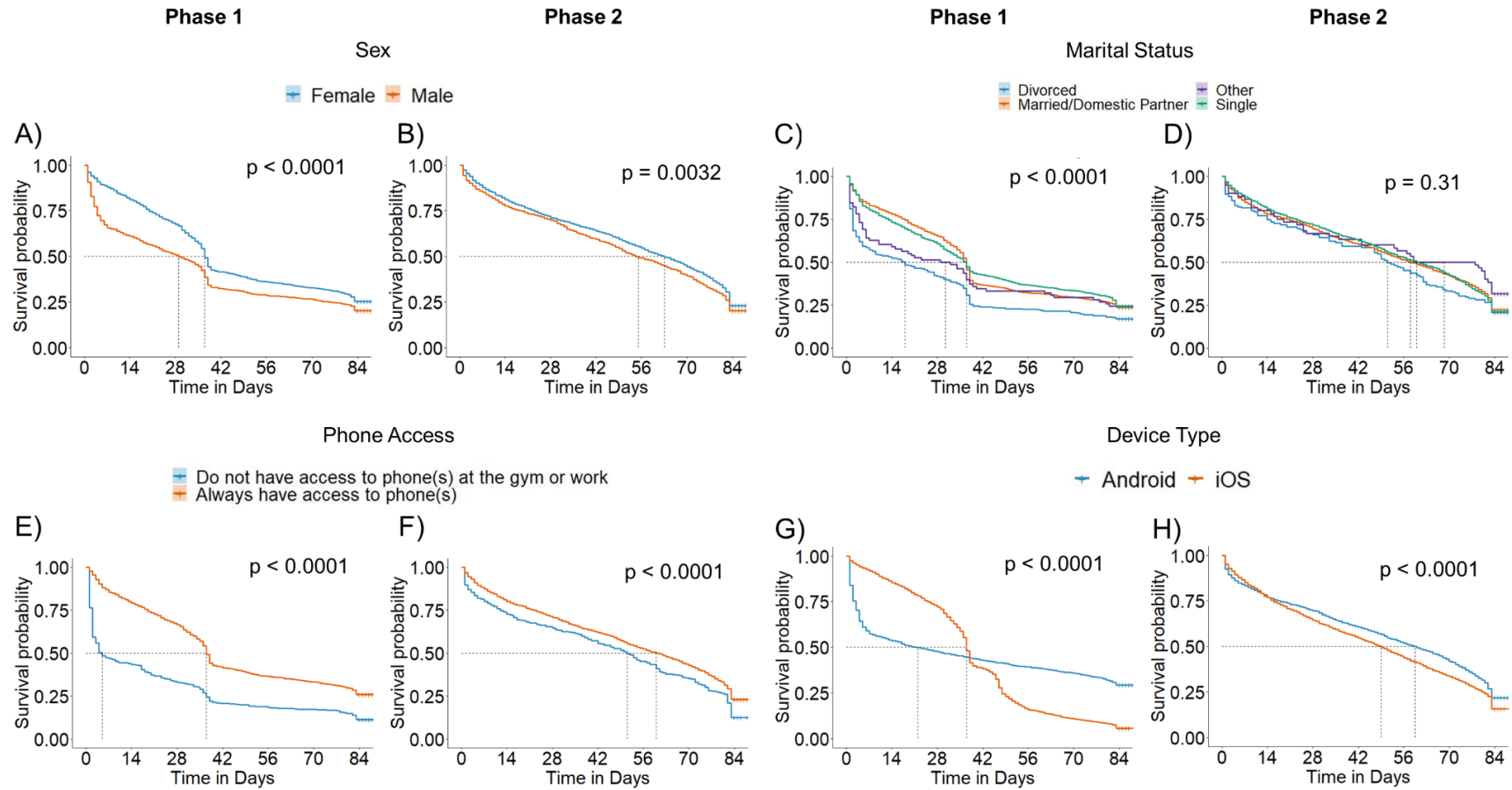

Supplement: Multimedia Appendix 8 [file formative_v6i11e40765_app8.pdf]
