# Supplementary material for: Recruitment and Retention in Remote Research: Learnings From a Large, Decentralized Real-world Study
Source: JMIR Form Res. 2022 Nov 14;6(11):e40765. doi: 10.2196/40765 (PMC9706389; doi:10.2196/40765)

Multimedia Appendix 7 - Sensitivity analysis of participant retention by extending the observation window (84 days) by 2 weeks

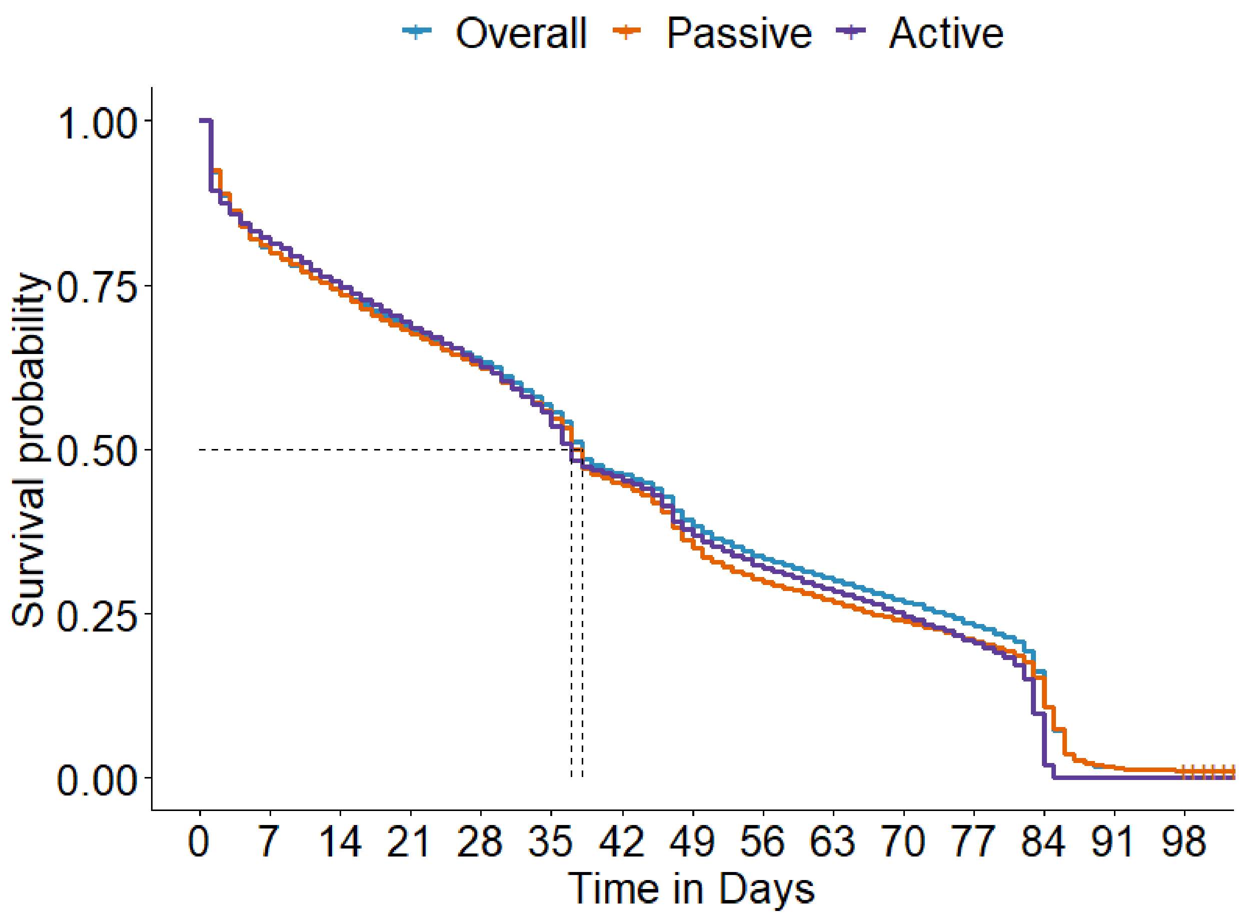

Supplement: Multimedia Appendix 7 [file formative_v6i11e40765_app7.pdf]
