# Supplementary material for: Recruitment and Retention in Remote Research: Learnings From a Large, Decentralized Real-world Study
Source: JMIR Form Res. 2022 Nov 14;6(11):e40765. doi: 10.2196/40765 (PMC9706389; doi:10.2196/40765)
Supplement: Multimedia Appendix 9 [file formative_v6i11e40765_app9.pdf]

Multimedia Appendix 9 - Patterns in Study App Data Collection

In both phases, the proportion of participants sharing passive data each day was generally higher than participants sharing active data (Phase 1 Median $_{\text{active vs passive}}$: 70.7% and 84.8%; Phase 2 Median $_{\text{active vs passive}}$: 56.6% and 71.25%) (a). However, data collection in Phase 2 showed higher day-to-day variation across active and passive data streams (b). Specifically, two periods (March 06 - April 14, 2021, and November 02 - December 06, 2021) were observed when the study app collected no passive data despite having participants complete active tasks. This indicates a likely technical glitch in passive data collection.

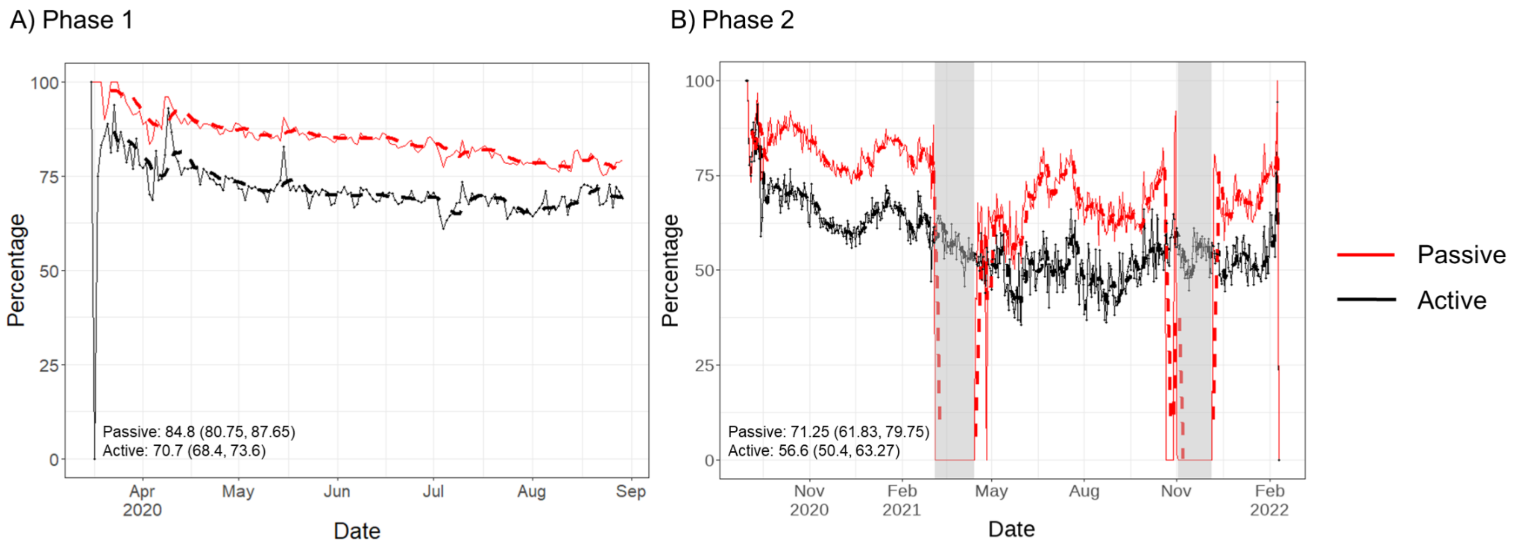

The relative proportion of participants who contributed data on any given day is faceted by a.) Phase 1 and b.) Phase 2 Black and red lines represent active and passive data, respectively. The dotted lines represent the seven-day moving average. Gray shaded areas in (b) show the study periods when no passive data was collected.
